# The Effect of High-Pressure Hydrostatic Extrusion on Mechanical Properties of Printed with Fused Deposition Modeling PLA and PLA-Diatomaceous Earth Composites

**DOI:** 10.3390/ma18030683

**Published:** 2025-02-04

**Authors:** Adrian Dubicki, Mariusz Kulczyk, Karol Szlązak, Maciej Łojkowski, Krzysztof Jan Kurzydłowski

**Affiliations:** 1 Faculty of Mechanical Engineering, Bialystok University of Technology, Wiejska 45C, 15-351 Bialystok, Poland; a.dubicki@pb.edu.pl (A.D.); maciej.lojkowski@pb.edu.pl (M.Ł.); 2 Institute of High-Pressure Physics, Polish Academy of Science, Sokolowska 29, 01-242 Warsaw, Poland; mariusz.kulczyk@unipress.waw.pl; 3 Faculty of Materials Science and Engineering, Warsaw University of Technology, Woloska 141, 02-507 Warsaw, Poland; karol.szlazak@pw.edu.pl

**Keywords:** PLA, 3D-printing, porosity reduction, diatomaceous earth, compressive strength, hydrostatic extrusion

## Abstract

Three-dimensional printing enables rapid prototyping, customization, and on-demand production. Polylactide is a popular biopolymer filament used in 3D printing. However, due to its brittleness and low mechanical strength, it often needs to be reinforced with filler particles. Diatomaceous earth shows great potential as a filler material due to its abundant and natural occurrence, biocompatibility, and environmental friendliness, as well as its excellent mechanical properties. Cold hydrostatic extrusion was used to improve the compressive strength of 3D-printed parts. Both neat and reinforced with 10% diatomaceous earth filaments were used to 3D print cylindrical billets, followed by post-processing using hydrostatic extrusion. X-ray microtomography showed a significant reduction in total and open porosity and average pore size, from ~20 µm to less than 10 µm in the Polylactide (PLA) and Diatomaceous (DE) composite. Compression tests showed a significant improvement in the compressive strength of PLA from ~60 MPa to ~100 MPa, while PLA with DE achieved an impressive almost twofold increase to 80–120 MPa. This was attributed to a reduction in pore size, as well as pore closure, which mitigates crack initiation in semi-brittle PLA. In addition, it has been proposed that hydro extrusion-induced structural rearrangement is an important strengthening factor.

## 1. Introduction

In recent years, polylactide (PLA) has attracted significant interest as a biodegradable biopolymer due to its versatility, flexibility, and ease of processing [1,2,3,4].

Products made of PLA are widely fabricated using injection molding [5], which can also be used for PLA composite materials, such as PLA with added recycled carbon fibers [6]. More recently, PLA composites have been used for 3D printing with Fused Deposition Modeling (FDM). An extensive review of the FDM 3D printing of PLA composites with biomass can be found in Bhagia et al. [7].

However, PLA applications are somewhat limited because of their relatively low mechanical strength. PLA’s poor mechanical performance stems from its brittleness at room temperature, low crystallization rates, low glass transition temperature (~60 °C), and limited thermal resistance. These properties restrict its use in applications requiring high-impact resistance or load-bearing capabilities, such as structural components or machinery parts [8,9,10]. Applications in high-temperature environments are also limited, as in the automotive or electronic industries [11,12].

Multiple strategies have been developed to address this problem. Among others, these strategies involve the addition of plasticizers and strengthening particles [13]. Furthermore, approaches such as incorporating nucleating agents, chain extenders, or branching chains have been explored to enhance crystallization rates [9,14].

Blending PLA with other polymers and compatibilizers can improve the ductility and mechanical strength in some cases, making PLA a suitable degradable material for bone tissue regeneration. Hue et al. have reported a tensile strength of 55 MPa for a PLA/Poly(Methyl Methacrylate) (PMMA) /Polycaprolactone (PCL) blend with substantial elongation to break increase [15].

However, there is still a need for an effective large-scale manufacturing process for PLA mechanical strength enhancement.

Diatomaceous earth (DE) is a promising “green” filler component for polymer/powder composites. DE is derived from the fossilized remains of microscopic aquatic organisms called diatoms, with silicon dioxide as its primary component. It is inherently porous due to the complex microscopic structure of diatom shells. One of its key advantages is that it occurs naturally and is abundant, making it both cost-effective and environmentally friendly [16,17,18].

PLA/DE composites have been investigated for their potential to improve thermal and mechanical properties. It was reported that Young’s modulus and impact strength of PLA/DE composites improved, while tensile strength and elongation at break either decreased or remained similar, depending on the DE particle size [19]. Similarly, a decrease in tensile strength and elongation at break, accompanied by an increase in Young’s modulus, was observed as a function of DE weight fraction [20].

Moreover, PLA/DE composites have been reported as suitable filaments for 3D printing. It was concluded that the addition of DE does not induce significant material degradation after 3D printing and is appropriate for applications requiring moderate mechanical endurance, while also potentially reducing filament costs by 5–10% [21]. However, consistent with the aforementioned studies, a decrease in mechanical properties was reported. The decrease in the mechanical properties was associated with an increase in the overall porosity, introduced by the DE addition.

This indicates that the PLA/DE composite is a promising material for 3D printing. However, further processing is needed to fully unlock its potential.

Both 3D printing and the addition of strengthening particles introduce porosity into PLA parts. These process-induced pores can significantly impact the properties of load-bearing polymeric parts at temperatures below the glass transition, where polymers exhibit semi-brittle behavior [22].

In this study, we propose improving the mechanical properties of PLA parts by utilizing cold-hydrostatic extrusion (HE). To date, few studies have investigated the effect of HE on the mechanical properties of PLA/DE composites. Furthermore, to the best of our knowledge, we are the first to report on the effect of HE on FDM 3D-printed PLA composite parts in which DE particles are used as a strengthening agent.

HE is a well-established process utilized in many applications, including high-performance components for automotive and aerospace industries, as well as for the processing of biodegradable polymers for packaging and medical applications [23]. It is known for improving the mechanical properties by changing the molecular orientation and structural alignment in polymers, resulting in enhanced tensile strength [24]. HE is used to obtain severe plastic deformation (SPD) to alter the grain structure. SPD transforms more coarse grains into fine-grain structures. This transformation limits the crack propagation within the materials and maintains the materials’ cohesion even at very high strains [25].

It was found that HE is suitable for processing brittle polymers by reducing stress concentration and improving ductility during the process. Moreover, HE was reported to provide smoother surfaces and better dimensional accuracy due to uniform pressure distribution and deformation [26]. HE was reported to allow optimization of extrusion parameters, like temperature, pressure, and strain rate, with high precision [27].

Attempts to apply HE to improve polymeric materials’ properties had already been undertaken in the late 70s of the 20^th^ century. Yoji Maeda et al. [28] studied extruded polypropylene (PP) with an extrusion ratio in the range of 2.0 to 6.5 at temperatures of 90–150 °C. Comprehensive structural characterization of the extruded material revealed changes in the degree of crystallinity in the processed PP.

HE was found to increase the strength of PLA parts by inducing macromolecular alignment along the extrusion axis. The effect of HE on the properties of poly-L-lactide (PLLA) was reported by Maeda et al. [28]. In this study, the billets were heated to temperatures ranging from 130 to 165 °C, and the reduction in the cross section varied from 3 to 12. It has been demonstrated that such extrusion conditions bring about significant changes in the mechanical properties of the processed billets. An increase was particularly high in the case of flexural stress, reaching 350 MPa. A significant increase in strength was observed in comparison to injection molded analogs. This increase was attributed to the change in macromolecular alignment. This effect is expected in the case of extrusion at temperatures approaching or exceeding glass transition and for a larger reduction in extruded billet diameter.

An investigation of Polyamide 6 (PA6) subjected to HE has shown great improvement in the tensile strength and tensile modulus. However, a slight reduction in compression strength was discovered. A structural change was found. The coarse grains in PA6 were transformed into highly oriented fibrous structures along the extrusion axis [29]. On the other hand, adiabatic heating during the process has caused partial melting of the crystalline phase and, combined with high strains, has led to increased porosity. An increase in crystallinity and transformation of crystallite morphology in PA6 subjected to HE was also reported [30].

Here, we have investigated the effect of the addition of DE to PLA combined with HE for reinforcing the FDM printed parts. We aimed to improve the compressive strength of the 3D-printed PLA/DE composite parts. We intended to combat the drawbacks of 3D printing the PLA parts and the decrease in mechanical properties due to DE addition. Moreover, it is worth noting that in the current study, we have departed from the molded-induced symmetry. The samples were printed such that each filament layer was arranged in a continuous spiral configuration. The mechanical strength of the components was investigated using compression tests. The change in porosity resulting from HE treatment was investigated using X-ray microtomography (micro-CT). The increase in the compressive strength was discussed with respect to the change in the pore morphology. In contrast to previous studies, we used cold HE to prevent excessive melting.

## 2. Materials and Methods

### 2.1. Three-Dimensional Printing Filament Preparation

Two types of filaments were used to fabricate 3D printed parts: (a) commercially available PLA (BASF, Ludwigshafen, Germany) and (b) PLA-based composite modified with 10 w/w% of DE (PERMA GUARD, Otwock, Poland) and prepared in a three-stage procedure route consisting of the following:

1. Intensive mixing of DE with PLA at 200 °C

2. Granulation

3. Extrusion using Friend Machinery FLD25 operated at 170 °C.

The DE PERMA GUARD contains primarily bio-silica shells of Aulacoseira cf. subarctica. The specific density of this DE is 2.2135 g·cm^3^, and the average size of particles in DE, as measured by the laser sizer, is 17.8 µm ± 0.2 µm. BET surface area is 32.7 m^2^·g^−1^. In addition to bio-SiO_2_, DE contains oxides of Al, Mg, Fe, and Ca. In the context of using DE as a modifier of polymeric matrices, it should be noted that the DE shells feature intricate topography with regularly spaced openings. These openings have sub-micron size and appear in a local density of 0.5/µm^2^.

### 2.2. Hydro Extrusion

Cylindrical parts were printed with FLASHFORGE CREATOR PRO2 (Sygnis, Gdańsk, Poland) with a 0.4 mm nozzle heated to 200 °C. The height of the layers was 0.2 mm. The billets, in the form of cylinders, had a diameter of 16 mm and a length of 150 mm.

**Figure 1:**
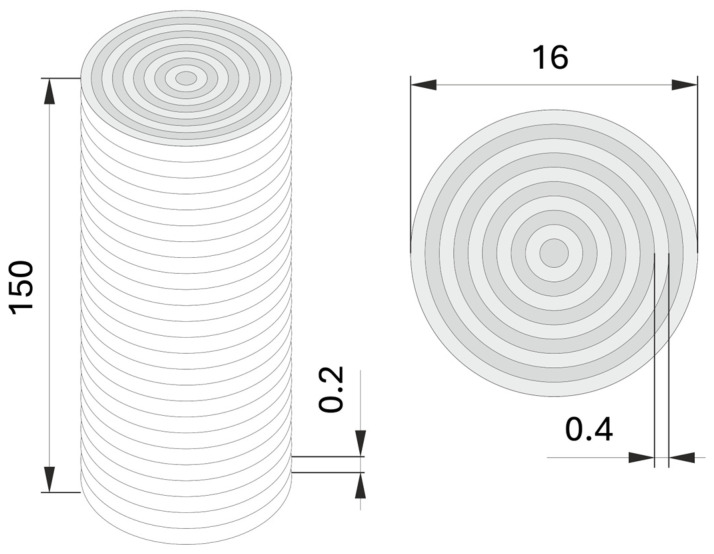
Explanation of printing strategy adopted for the fabrication of the billets. The billets were printed layer by layer with every layer consisting of concentric circles.

FDM printing profoundly transforms filament symmetry into a centripetal-lamellar structure. The billets were printed layer by layer with the axis perpendicular to the support plate. Each layer, 0.2 mm thick, was printed by fusing concentric circles of diameters diminishing to a spot in the center of the billets—as schematically shown in Figure 1.

The printed cylinders were HE at the Institute of High-Pressure Physics of the Polish Academy of Sciences in a single step with a diameter reduction to c.a. 11 mm. This resulted in the reduction in cross section equal to 3.1 mm. The die diameter was 9 mm. The extruded material was intensively cooled in a flow of cold water at the exit of the shaping die. Neither the billet nor the extruder had been heated prior to the extrusion. Equivalent strain induced by extrusion can be estimated as equal to 1.15. The pressure applied exceeded 150 MPa. The extrusion process of one billet lasted approximately 20 s of stable pressure period.

### 2.3. Density, Microstructure, and Mechanical Properties

The density of the samples was measured using the Archimedes method in deionized water and dehydrated ethanol, using a XS204 analytical balance (Mettler Toledo, Greifensee, Switzerland) with an XPR/XSR density measurement kit. The tests used 3D-printed samples and those after the HE process. The measurements were carried out at a liquid temperature of about 22.5 °C, for which the density of dehydrated ethanol, according to the tables, is 0.78720 g/cm^3^, and distilled water, 0.99768 g/cm^3^.

### 2.4. X-Ray Microtomography Tomography

Microstructures of the as-printed and printed HE specimens have been investigated using X-ray micro-CT. The prepared samples were examined by means of Xradia XCT-400 (Zeiss, Oberkochen, Germany). The X-ray tube voltage was 40 kV, and the current was 250 µA. The X-ray projections were obtained at 0.18° intervals with scanning angular rotation of 180°. The obtained pixel size was 2.2 µm. Observations were focused on pores that exist in composite filaments and 3D-printed cylinders.

### 2.5. Compression Testing

Samples for compression tests were machined out of the HE-processed billets in the form of cylinders with a length 9 mm and a diameter 6 mm. Control samples were printed using FDM technology of the same dimensions. 858 Mini BIONIX (MTS Systems, Eden Prairie, MN, USA) testing machine with ARAMIS 3D 4M vision system, (GOM Correlate software 2018, GOM GmbH, 2018, Zeiss, Oberkochen, Germany) was used for straining operated at room temperature. The initial strain rate was 1.85 × 10^−3^ s^−1^.

## 3. Results

### 3.1. Hydro Extrusion Pressure

**Figure 2:**
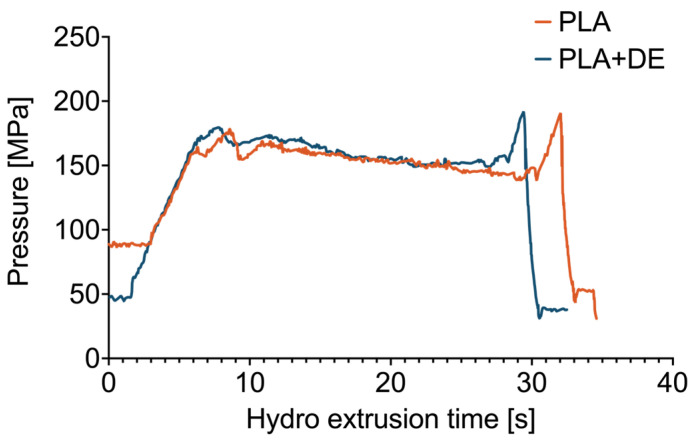
Changes in pressure values as a function of time during the hydro extrusion process.

Changes in pressure values as a function of time during the hydro extrusion process are shown in Figure 2.

The initial increase in pressure is related to the need to overcome frictional forces on the shaping die. Further along in the deformation process, the pressure stabilizes. Minor pressure oscillations may be related to thermal effects occurring in the deformation zone, resulting in the observed character of the product surfaces. A comparison of pressure runs for the two materials, PLA and a PLA+DE composite, show no significant differences in the strengthening during plastic processing. In contrast, the differences between the samples would signalize differences in the strain and deformation of the samples [29]. Here, the true strain was the same for both kinds.

### 3.2. X-Ray Micro-CT of Filaments Microstructure Before Hydrostatic Extrusion

**Figure 3:**
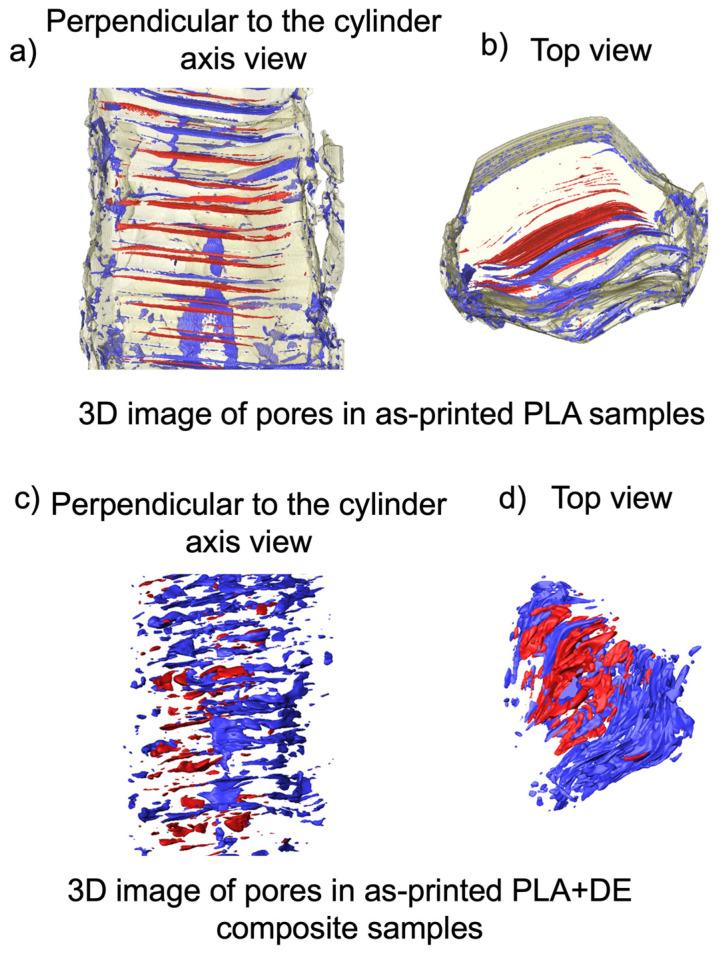
Reconstruction of the porosity for as-printed billets based on the data obtained from the micro-CT. (**a**,**b**)—as-printed PLA 3D samples; (**c**,**d**)—as-printed PLA+DE 3D samples. The color marking corresponds to the porosity type. Open pores are marked blue, while the closed pores are marked red.

X-ray scans have been used to reconstruct 3D microstructure features and determine their porosity. Scanning was performed on pieces cut off from the respective samples with a size of approximately 2.2 mm^3^. The small size of the samples assured high-resolution imaging. Results of the reconstructions for as-FDM printed samples are presented in Figure 3.

In the case of as-printed samples (PLA 3D, Figure 3a,b), quantitative analysis of the pores showed that the total porosity is equal to 1.1%, with the open pores accounting for 0.8%. The size of individual pores, as determined by fitting maximal spheres to every point in the structure [31], is similar for closed and open ones and ranges up to 40 µm and 70 µm, respectively, with the highest experimental density between 5 µm and 10 µm and the average size being 17 µm for both.

In the case of as-printed PLA with DE composite samples (PLA+DE 3D), quantitative analysis of the pores (Figure 3c,d) showed that the total porosity is equal to 6.0%, with the open pores accounting for 4.3%. The size of individual pores is similar for closed and open ones and ranges up to 70 µm, with the highest experimental density between 5 µm and 10 µm, and the average size being 23 µm and 22 µm, respectively.

It can be noted that FDM-printed composite samples have significantly higher porosity. This is because of gas micro-bubbles being entrapped in the fabrication of the composite filament. In fact, a micro-CT showed that its porosity reached 6.9%, with practically all pores being closed. Thus, FDM printing simply transforms the closed porosity of the composite filament into predominantly surface pores.

Reconstructed 3D micro-CT microstructures reveal that the pores are elongated and nearly perpendicular to the axis of the cylinders.

### 3.3. X-Ray Micro-CT of Filaments Microstructure After Hydrostatic Extrusion

**Figure 4:**
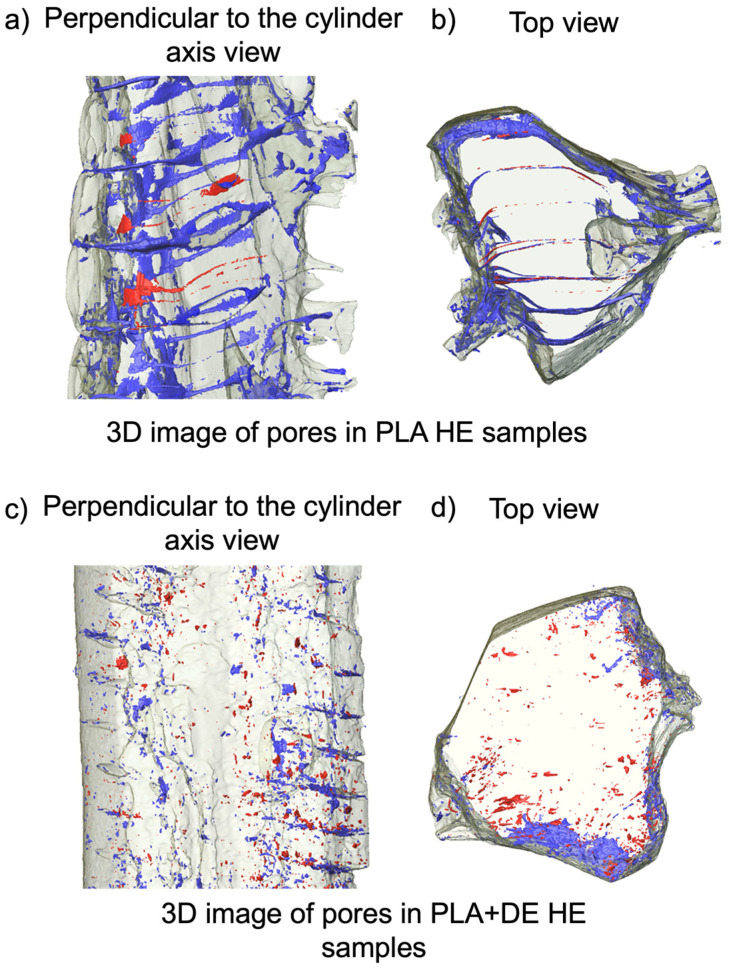
Reconstruction of the porosity for HE post-processed billets based on the data obtained from the micro-CT. (**a**,**b**)—PLA 3D HE samples; (**c**,**d**)—PLA+DE 3D HE samples. The color marking corresponds to the porosity type. Open pores are marked blue, while the closed pores are marked red.

Results of the reconstructions for FDM printed samples after HE are presented in Figure 4.

In the case of PLA samples subjected to HE (PLA 3D HE, Figure 4a,b), quantitative analysis of the pores showed that the total porosity is below 1%, with the open pores accounting for c.a. 0.06%. This indicates a substantial reduction in closed pores, also in size with an average close to 11.5 µm.

In the case of the composite samples subjected to HE (PLA+DE 3D HE, Figure 4c,d), the reduction in the porosity is even higher. Quantitative analysis of the pores showed that the total porosity is c.a. 0.5%, with the closed pores accounting for 0.2%. This indicates the substantial reduction in closed pores, and size, with an average below 10 µm. All porosities, as determined experimentally by micro-CT, are listed in Table 1.

It was observed that the type of porosity changed after HE compared to the printed samples. In the pristine PLA samples, the open pores, initially perpendicular to the main axis, transformed into closed pores. Moreover, these pores became twisted and were no longer perpendicular to the main axis.

On the other hand, the PLA+DE samples subjected to HE were significantly densified. The PLA+DE samples before HE had very large porosity of 6%, which decreased twelve times to 0.5% after processing. Only very small, isolated pores were found within the bulk of the sample, while the remaining porosity was concentrated at the edges.

In summary, HE of the FDM printed samples results in a profound reduction in the total porosity and volume fraction of closed pores. This reduction is particularly high in the case of composite samples.

### 3.4. Structural Size Parameter

**Figure 5:**
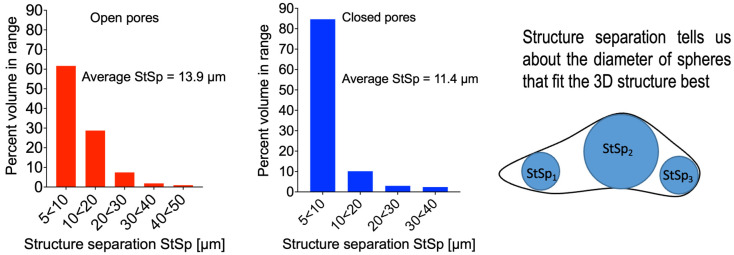
Schematic explanation of the procedure for assigning size parameters to individual pores.

Based on the results of X-ray CT, the size of pores has been determined using the parameter explained in Figure 5, which also shows experimental size distribution for open and closed pores in PLA after hydro-extrusion. The average values of the Structural Size Parameter (StSp) are listed in Table 2.

It can be noted that HE brings about a significant reduction in the size of pores. The reduction in size is particularly impressive in the case of PLA+DE composite samples, from over 20 μm to less than 10 μm.

### 3.5. Density of the FDM Printed Samples

The density measurement results are presented in Table 3. The tests were conducted on pieces of material up to 15 mm in size, cut from individual samples.

The values obtained from density measurements in ethanol are higher than in water. This is due to the better wettability of the sample surface with open porosity by ethanol. Samples that were 3D printed, in general, show an increase in density after HE. As with agreement to the X-ray micro-CT porosity analysis, the highest density increase is observed for PLA+DE 3D HE samples.

### 3.6. Compression Tests

**Figure 6:**
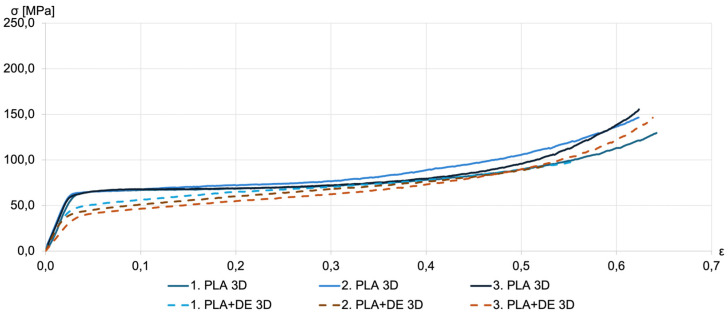
Representative compression stress–strain curves for PLA 3D (solid lines) and PLA+DE 3D as-printed samples (dashed lines). The multiple lines shown in the figure for each kind correspond to repeated measurements.

**Figure 7:**
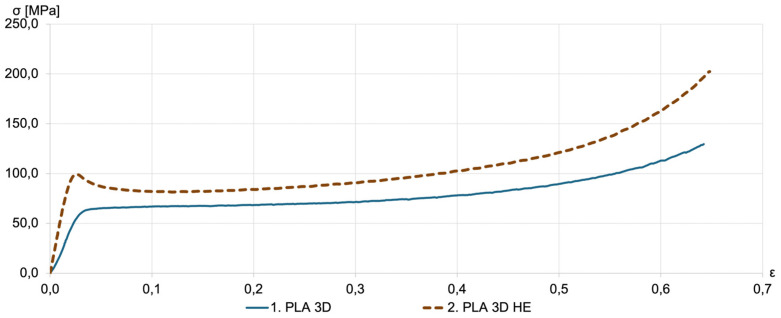
Representative compression stress–strain curves for as-printed PLA 3D samples (solid line) and subjected to HE after printing (PLA 3D HE, dotted line).

**Figure 8:**
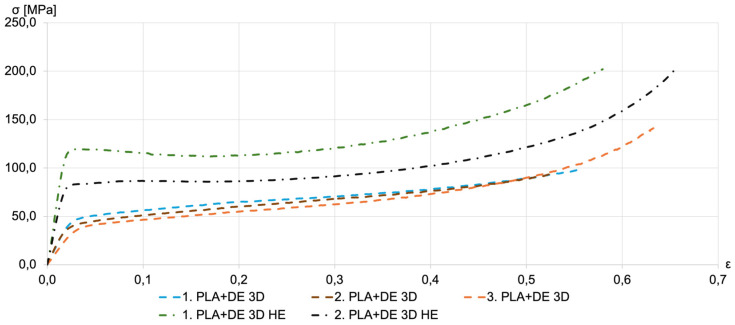
Representative compression stress–strain curves for printed PLA+DE 3D samples before extrusion (dashed lines) and for extruded PLA+DE 3D HE samples (dashed-dotted lines). The multiple lines shown in the figure for each kind correspond to repeated measurements.

**Figure 9:**
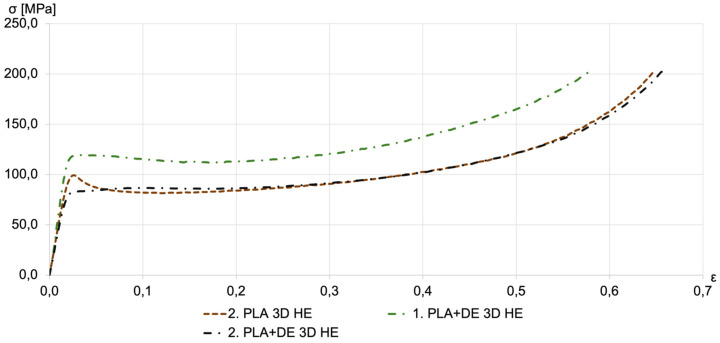
Compression stress–strain curves for PLA and PLA+DE samples subjected to HE. Representative compression stress–strain curves for printed PLA+DE 3D samples before extrusion (dashed lines) and for extruded PLA+DE 3D HE samples (dashed-dotted lines). The multiple lines shown in the figure for each kind correspond to repeated measurements.

Results of the compression tests are shown in Figure 6, Figure 7, Figure 8 and Figure 9 in the form of compression stress–strain curves. Figure 6 illustrates the compression strength of as-printed specimens fabricated by FDM using a PLA filament and the filament with 10% DE.

For as-printed PLA samples, the yielding is observed at a compression stress of approximately 60 MPa. This agrees with values reported for PLA samples fabricated with the FDM method—see, for example, Vukasovic et al. [32]. It can be noted that the composite samples PLA+DE have lower compression strength in the range of 40–50 MPa. This can be explained by the higher porosity of samples printed with the PLA+DE filament.

The effect of HE on as-printed PLA without DE samples is shown in Figure 7. A significant increase in the compressive strength was observed. The compressive strength exceeded 100 MPa. This implies a 60% increase in compression strength in the case of samples printed with a PLA filament.

Stress–strain curves for the 3D-printed PLA with DE (PLA+DE 3D) samples, both before and after HE, are shown in Figure 8. HE treatment significantly enhanced the compressive strength of the samples. Notably, the HE-treated PLA+DE samples also demonstrated substantial improvements in compressive strength compared to those without DE. Despite the relatively large starter of the measurement results, the compressive strength of HE-treated PLA+DE samples ranged between 80 and 120 MPa.

Figure 9 compares the compressive strength of hydro-extruded PLA and PLA+DE composite samples. The stress–strain curve for PLA exhibits a distinct “yield point”, which is absent in the curves for PLA+DE samples. The compressive strength of samples printed with both filaments consistently exceeds 80 MPa.

It should be noted that the values of compression strength obtained in the current study agree with the reported by Gao et al. [33]. The compression tests carried out by these authors on printed PLA samples showed compression stresses in the range of 48–62 MPa, depending on the additions used to obtain the desired color of the samples.

Similar values have been reported by Zglobicka et al. [20] who investigated composites with 10 w/w % of DE based on different types of PLA. They reported tensile strength in the range of 48–67 MPa and elastic modulus in 3.3–4.4 GPa. The literature data indicate E = 3.5 GPa as the most measured value of elastic modulus—see, for example, Farah et al. [8].

Based on comprehensive studies of the properties of 3D printed specimens, Claudio et al. reported the following maximum values for compressive modulus and yield stress: 2.9 GPa and 69.5 MPa, respectively [34].

## 4. Discussion

Strain-induced degree and spatial orientation of the alignment of macro-molecules depend on the geometrical transformation involved in the processing in question. In the current study, four operations can be distinguished which induce re-orientation of the macro-molecules:

1. Uniaxial alignment in the fabrication of the PLA+DE filament

2. Uniaxial alignment during hot extrusion Via dye of printer—PLA and PLA+DE

3. Planar-circular alignment during FDM printing

4. During hydro-extrusion of printed billets

In stages 1 and 2, macromolecules are expected to become aligned along the filament. In stage 3, they assume the orientation of circular rings perpendicular to the axis of the billet. Finally, in stage 4, the arrangement of macromolecules is changed by the reduction in the billet’s diameter.

In the current study, the closure of closed pores was found to be the major effect of HE on processed billets. In the case of PLA 3D samples, total porosity is below 1.1% and remains practically unchanged during hydro-extrusion, which increases open porosity slightly and reduces closed porosity below 0.1%. With no addition of strengthening particles, an increase in compression strength is, therefore, related to an increase in the strength of the polymeric matrix. Since in the current study, we applied so-called cold extrusion, the latter can be explained as caused by the change in the macromolecular alignment [29,30].

**Figure 10:**
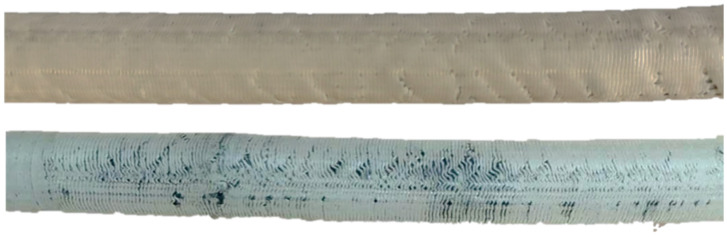
Photographs of the extruded billets.

In general, there are two conceivable modes of transforming the rings/tori in the billets during the extrusion-induced reduction in diameter. First, the ring/tori may assume corrugated geometry. The second option is rotation against the axis perpendicular to the axis of the cylinder, transforming circular rings/tori into ellipsoidal. The surface topography of the extruded billets (Figure 10) and geometry of pores (Figure 3) indicate that both modes of re-alignment are taking place, jointly causing specific strain hardening, manifesting itself in an increase in the compression stress of the extruded billets. Considering negligible changes in the porosity, the hydro-extrusion strengthening of printed PLA is impressive and calls for exploring its practical implications.

HE-induced reduction in porosity is particularly high in the case of composite samples PLA + DE. This is because, generally, a relatively high density of pores is typically observed in such composites because of technological constraints related to the dispersion of diatoms in the polymeric matrix.

However, the total porosity of 6% in PLA+DE samples, as compared with the 1% porosity of PLA samples, hardly justifies the difference in compression strengths, 40 and 60 MPa, respectively. A simple rule of mixing would suggest that the difference should be approximated by 5% of the compression strength of printed PLA, i.e., 3 MPa, as opposed to 20 MPa observed experimentally. Thus, it might be concluded that the rule of mixing does not apply in this case.

The failure to apply the rule of mixing explains the current results as it is related to the semi-brittle properties of PLA at room temperature. Because of this brittleness, we suggest the need to investigate the size of pores in addition to the porosity (Table 3). The porosity was reduced from 20 μm to less than 10 μm.

The basic laws of fracture mechanics predict that the failure stress is inversely proportional to the square root of the size of flaws (Equation (1)):

(1) $$\sigma =K\sqrt{c}$$

where *σ* is failure stress, K stands for material constant, and c is the size of the critical flaw. Assuming proportionality between c and the average size of pores, one can estimate that the two-fold decrease in the size of flaws induced by hydro extrusion of PLA+DE samples should increase failure stress from 40 MPa to about 60 MPa, in excellent agreement with the experimental observations. It should be added that for PLA printed samples, the reduction pore size is much lower, particularly low in the case of open pores, which account for 96% of the total volume of pores.

## 5. Conclusions

It has been successfully demonstrated that hydrostatic extrusion is a highly effective method for increasing the compressive strength of 3D printed parts. An impressive increase in the compressive strength of printed PLA parts was observed. The compressive strength increased from 60 MPa to about 100 MPa.

In addition, the compressive strengthening by HE of 3D printed parts made of PLA with 10% DE addition was investigated. A significant improvement in the compressive strength, reaching 80–120 MPa, was recorded. The application of HE processing helped to overcome the initial strength loss caused by increased initial porosity in the 3D-printed neat and composite samples.

Micro-CT analysis showed that HE dramatically reduced total and open porosity in both pure PLA and PLA+DE samples. It is worth noting that the porosity of PLA+DE dropped from about 6.0% to just 0.5%. Along with the reduction in overall porosity, HE reduced the average pore size from ~20 µm to less than 10 µm. This reduction in the size of potential crack initiation sites is crucial for improving mechanical strength, especially in semi-brittle PLA.

The above discussion of the results leads to the conclusion that the effect of HE on the compressive strength of the tested samples can be explained by a reduction in both total porosity and, most importantly, pore size.

Overall, the combination of DE and cold hydrostatic extrusion solves two disadvantages of PLA composite 3D printing parts. It reduces the porosity introduced by both the introduction of the DE particle filler and 3D printing itself. Furthermore, it combats PLA’s natural brittleness by improving its load-bearing capability.

This work paves the way for future exploration of combining the flexibility of 3D printing, which allows for the creation of diverse structural architectures using PLA/DE filaments with HE post-processing. The combination of the mentioned methods could combat the inherent room temperature brittleness of PLA. However, more comprehensive structural and mechanical characterization is needed. Future work could also discuss the incorporation of plasticizers and/or crystallizing agents to further explore the PLA potential to produce high-performance PLA parts.

## Figures and Tables

**Table 1:** Porosity as measured from micro-CT observations.

Specimen	Porosity [%]		
	Open	Closed	Total
**PLA 3D**	0.8	0.3	1.10
**PLA 3D HE**	0.9	0.06	0.96
**PLA+DE 3D**	4.3	1.7	6.00
**PLA+DE 3D HE**	0.3	0.2	0.50

**Table 2:** The average value of StSp.

Specimen	Average Size [μm]	
	Open	Closed
**PLA 3D**	17.2	16.6
**PLA 3D HE**	13.9	11.4
**PLA+DE 3D**	22.9	21.8
**PLA+DE 3D HE**	10.4	9.9

**Table 3:** The density of samples as printed and subjected to HE.

Specimen	Density [g/cm3]	
	In Water	In Ethanol
**PLA 3D**	1.1913	1.1845
**PLA 3D HE**	1.2167	1.2353
**PLA+DE 3D**	1.1790	1.1805
**PLA+DE 3D HE**	1.2347	1.2613

## Data Availability

The data supporting this study’s findings are available from the corresponding author upon request.

## Abbreviations

The following abbreviations are used in this manuscript:

DE	Diatomaceous Earth
FDM	Fused Deposition Modeling
FLD	Filament Extruder Machine
HE	Hydrostatic Extrusion
MTS	Material Testing System
PA6	Polyamide 6
PLA	Polylactic Acid
PLLA	Poly-L-Lactic-Acid
PP	Polypropylene
SPD	Severe Plastic Deformation
micro-CT	X-ray computed microtomography
